# Editorial: Therapeutic drug monitoring and clinical toxicology of anti-cancer drugs

**DOI:** 10.3389/fonc.2022.1053211

**Published:** 2022-10-25

**Authors:** Yao Liu, Jennifer H. Martin, Miao Yan

**Affiliations:** ^1^ Department of Pharmacy, Daping Hospital of Army Medical University, ChongQing, China; ^2^ School of Medicine and Public Health, University of Newcastle, Newcastle, NSW, Australia; ^3^ Department of Pharmacy, the Second Xiangya Hospital of Central South University, ChangSha, Hunan, China; ^4^ Hunan Provincial Center for Poison Information and Treatment, ChangSha, Hunan, China; ^5^ International Research Center for Precision Medicine, Transformative Technology and Software Services, Hunan, China

**Keywords:** therapeutic drug monitoring, clinical toxicology, anti-cancer drugs, editorial, precision medicine

## Introduction

Cancer incidence in China is currently the highest in the world, and the demand for antineoplastic drugs is thus also growing. In the context of precision medicine and precision pharmaceutical services, anti-tumor drugs have transitioned from traditional chemotherapy drugs to combined use with molecular targeted drugs and immunosuppressants. Many of these have come into clinical practice rapidly, with recommendations to use a single dose despite significant inter-individual variability in achieved exposure between patients.

In addition, most of these antineoplastic drugs have the characteristics of a narrow therapeutic window, large individual differences in drug metabolism, nonlinear pharmacokinetic characteristics and obvious organ toxicity particularly when exposure is above recommended target exposure ranges. Further, in order to achieve personalized medicine and precision medicine, factors such as the patient’s genes, enzymes, diseases, drug sensitivity to tumors, multi-drug combinations and patient characteristics need to be considered. **(** 1–3 **)**. Therefore, along with research into cancer biology and into the effects of these agents in different cancer genotypes or phenotypes, studies on therapeutic drug monitoring (TDM) and population pharmacokinetics (PPK)/pharmacodynamics (PD) for anti-cancer drugs play a crucial part in the optimization of antineoplastic regimens and precise drug treatment. These tools can minimize drug-induced toxicity and maximize the treatment outcome. In addition, although antitumor drugs have changed from conventional chemotherapeutic drugs with low selectivity and high toxicity to molecular targeted drugs with high selectivity and low toxicity, the related adverse events (AEs) involving vital organs, such as related cardiotoxicity, endocrine, gut and liver toxicity, cause significant morbidity and sometimes death **(** 4–7 **)**. The in-depth toxicological research of these newer therapies in the clinical setting is vital to better understand the factors contributing to toxic effects and provide guidance for improved drug use.

In this special issue, 24 manuscripts, including nine review articles and fifteen original research articles providing a wide discussion of TDM and clinical toxicology of antineoplastic agents in different clinical settings. This special issue thus aims to both provide guidance on more individualized drug administration plans for treatment using TDM and PPK/PD research and to understand the potential mechanism of antitumor drugs therapeutic and toxic side effects.

Firstly, there were some articles and reviews that explored and provided an update on research progress of PPK, PD and TDM in the response and toxicity of anti-tumor drugs. Wen et al. systematically reviewed the recent progress of PK-PD modeling in predicting cardiovascular adverse reactions and how to manage this in the clinical setting. He et al. summarized the existing evidence of the clinical PK variation of dasatinib concentration-response relationships and advice on development of methods for individualizing the dosage of administration. In addition, Barnett et al. provided a summary of current research using TDM in pediatric cancer and implementing TDM-based dosing recommendations. And Huang et al. built a new PK model of busulfan (BU) providing guidance for patients of Chinese descent to achieve individualized and optimal dosage regimens.

Then, plasma obtained by conventional venous blood sampling is usually standard matrix for TDM of antineoplastic drugs, with LC-MS/MS the conventional method of choice for measuring TDM. As an update, to measure 6-thioguanine and 6-methylmercaptopurine in red blood cells, Bajaj et al. used a LC-MS/MS method, evaluating the association between TM concentrations, thiopurine-S-methyltransferase (TPMT) phenotype and genotype testing. This provided a new approach for thiopurine TDM to minimize myelosuppression and the risk of hepatotoxicity. In addition, Guo et al. used HPLC-HG-AFS to analyze the concentrations of inorganic arsenic, methyl methacrylate (MMA) and nitrosodimethylamine (DMA) and used this to discuss features of intrauterine arsenic concentration, the permeability of the placenta to arsenic trioxide (ATO) and its metabolites and provided the first risk evaluation of ATO in pregnant women with acute promyelocytic leukemia. Verougstraete et al. summarized various analysis measuring methods of kinase inhibitors based on emerging dried blood microsample technique, which is minimally invasive and considered convenient and simple.

Third, there are also several systematic reviews and meta-analysis highlighting the toxicity and response of antineoplastic drugs. Zhang et al. identified the effectiveness and risk of adding capecitabine to the chemotherapy for triple negative breast cancer through a meta-analysis. Song et al. and Zhang et al. conducted both systematic review and meta-analysis to analyse the effects of genetic polymorphisms on the toxicity and response of high-dose methotrexate (HD-MTX) and response in tumor. In addition, Yang et al. conducted a pharmacovigilance study showing enfortumab vedotin (EV) was associated with severe skin toxicities. Meanwhile, based on the information from Henan Province’s spontaneous reporting system database, Jiao et al. analyzed potential organ toxicities in Chinese pediatric subjects, an area with a previous dearth of data. What’s more, Zhao et al. showed PARP inhibitors may induce myelodysplastic syndrome (MDS) and acute myeloid leukemia (AML), with some at higher risk than others. Yu et al. ***via*** a post-marketing surveillance research evaluated the risk of oxaliplatin (OXA) in 3687 Chinese cancer patients. Zhu et al. found SOAT1 can be a prospective prognostic indicator in gastric cancer and may help the clinical dosing regimen.

Besides, He et al. attempted to build a risk scoring model based on gene polymorphisms to predict adverse drug reactions caused by HD-MTX in children (age ≤ 16 years) especially hepatotoxicity based on various relevant indicators. This model informed the MTX regimen and using it reduced toxicity. Han et al. used retrospective and multicenter clinical data to establish a risk scoring system through machine learning methods for predicting liver injury with tyrosine kinase inhibitors. In general, by measuring drug exposure, pharmacological indexes or pharmacodynamic indicators in tumor patients, using technologies such as PPK, PD, TDM and databases to achieve individualized drug treatment these studies have provided individualized dosing regimens.

Meanwhile, in our special issue, 5 manuscripts described and summarized the potential molecular regulatory mechanisms of anti-tumor drugs to exert therapeutic effects and produce toxic side effects. Li et al. detailed the molecular pathways of kinase inhibitors induced EGFR-skin toxicity, strategies to attenuate severe skin toxicity, and provided information to manage such skin reactions. Yang et al. systematically reviewed the probable mechanisms, clinical features, diagnostic method, intervention measures and the most recent advancements in cardiotoxicity of ErbB2-targeting drugs, providing guidance for clinical practice. Han et al reviewed the current epidemiology, risk indicators, molecular regulation mechanisms, prevention and management of irinotecan-induced steatohepatitis, and Guo et al. showed that the kinase inhibitors crizotinib and sunitinib induced hepatotoxicity *via* oxidative stress and mitochondrial apoptosis pathways, suggesting Nrf2 might be a therapeutic target. Du et al. found that the inhibition of PRR could attenuate RAC1-NOX4 pathway and reduce ROS accumulation to weaken doxorubicin-induced heart failure, thus providing a prospective approach to the management of DOX-triggered heart failure. In general, these studies above have indicated the research actuality of anti-tumor drug-induced diseases and potential molecular regulatory mechanisms of toxicity.

Finally, in our special issue, there were also two manuscripts that explored risk management and effectiveness of PD-1/PD-L1 inhibitors. A case report (Tu et al.) showed that the use of avatrombopag in two cases of anti-PD-1 antibody-caused acquired megakaryocytic thrombocytopenia was successful, proposing a promising therapeutic option for this disease. Zhang et al. comprehensively summarized the molecular regulatory mechanisms of immune-related adverse events, the risk and therapeutic effect of PD-1/PD-L1 inhibitors administration in AID subjects, the prevention and control of organ toxicity and provides several promising treatment methods.

Taken together, this Research Topic contributes an update of the current clinical research into TDM and clinical toxicology of antineoplastic drugs to improve use of anti-tumor therapy, guiding clinical dose adjustment, and promoting the development of precision medicine.

## Author contributions

All authors listed have made a substantial, direct and intellectual contribution to the work, and approved it for publication.

## Funding

MY is supported by the National Natural Science Foundation of China, grant number 81974532 and 81803830, the Natural Science Foundation of Hunan Province, China, grant number 2020JJ4130.

## Acknowledgments

We wish to thank all the authors contributing to this Frontiers Research Topic and all the reviewers and invited editors who have helped to make it solid.

## Conflict of interest

The authors declare that the research was conducted in the absence of any commercial or financial relationships that could be construed as a potential conflict of interest.

## Publisher’s note

All claims expressed in this article are solely those of the authors and do not necessarily represent those of their affiliated organizations, or those of the publisher, the editors and the reviewers. Any product that may be evaluated in this article, or claim that may be made by its manufacturer, is not guaranteed or endorsed by the publisher.
